# Parental Education and Family Dissolution: A Cross‐National and Cohort Comparison

**DOI:** 10.1111/jomf.12461

**Published:** 2018-01-30

**Authors:** M.D. (Anne) Brons, Juho Härkönen

**Affiliations:** ^1^ Netherlands Interdisciplinary Demographic Institute and VU University Amsterdam; ^2^ Stockholm University

**Keywords:** cross‐national research, divorce, intergenerational, parental education

## Abstract

This is the first study to systematically analyze whether the association between parental education and family dissolution varies cross‐nationally and over time. The authors use meta‐analytic tools to study cross‐national variation between 17 countries with data from the Generations and Gender Study and Harmonized Histories. The association shows considerable cross‐national variation, but is positive in most countries. The association between parental education and family dissolution has become less positive or even negative in six countries. The findings show that the association between parental education and family dissolution is generally positive or nil, even if the association between own education and family dissolution is in many countries increasingly negative. The authors find suggestive evidence that the association is related to the crude divorce rate, but not to the generosity of the welfare state in these countries. The implications of these findings for understanding the stratification in family dissolution are discussed.

Advantaged family backgrounds pave the way to higher education, higher incomes, and better health (Breen & Jonsson, 2005; Elo, 2009). Higher socioeconomic backgrounds are also related to many favorable family demographic outcomes, such as postponement of childbearing beyond adolescence (Dahlberg, 2015) and marriage with highly educated partners (cf. Schwartz, 2013). Do favorable family backgrounds also beget family stability and the benefits associated with it? Recent research has paid much attention to the growing educational disparities in family dissolution (e.g., Amato, 2010; Härkönen & Dronkers, 2006; McLahanan, 2004), but this interest has not been matched by a similar focus on family dissolution patterns by parental educational background. Because of the importance of family background on individuals' future life chances, this omission limits our understanding of the social stratification in family demography.

Previous studies on the association between parental education and family dissolution have produced intriguing findings. In contrast to the increasingly negative association between own education and family dissolution in many societies (Härkönen & Dronkers, 2006), many studies have found a positive association between parental education and family dissolution (Sweden: Hoem & Hoem, 1992; the Netherlands: Klijzing, 1992; Janssen, 2001; Finnish women: Mäenpää & Jalovaara, 2014; Italy: Todesco, 2013), even when the relationship between own education and family dissolution is negative (Norway: Lyngstad, 2004, 2006). This suggests a nuance to perspectives of the lower status character of family dissolution that have come to dominate the literature on stratification of family instability. However, other studies reported zero relationships (Australia: Bracher, Santow, Morgan, & Trussel, 1993; United Kingdom: Berrington & Diamond, 1999; Finnish men: Mäenpää & Jalovaara, 2014) or a negative association (United States: Bumpass, Martin, & Sweet, 1991), suggesting that the association may vary cross‐nationally akin to the relationship between own education and family dissolution (Härkönen & Dronkers, 2006; Martin, 2006; Matysiak, Styrc, & Vignoli, 2014). Many of the above studies are also rather dated, raising the possibility that the association has changed over time, potentially from a positive to a negative one as has been reported for the educational gradient of divorce in many countries (Chan & Halpin, 2008; De Graaf & Kalmijn, 2006; Härkönen & Dronkers, 2006; Hoem, 1997; Raymo & Iwasawa, 2017).

This study presents the first comparative analysis of the association between parental education and family dissolution. In light of the previous discussion, we first ask whether parental education is related to family dissolution in 17 European societies and whether this association varies cross‐nationally. Second, has this association changed over time? Third, to understand the causes of the variation across societal contexts, we analyze whether cross‐national and cohort differences in the association can be linked to two contextual‐level variables that reflect the sociocultural and economic contexts of family life, namely, the average crude divorce rate and the generosity of the welfare state. Our analysis contributes to the understanding of (variation in) stratification of family dissolution and of intergenerational effects on family dissolution, other family demographic behaviors (Dahlberg, 2015; Dronkers & Härkönen, 2008; South, 2001; Wiik, 2009; Wolfinger, 2003), and life chances more generally. We use family history data from the Generations and Gender Study (GGS) and Harmonized Histories data sets. Our outcome is the dissolution of first childbearing unions, which is more suitable than divorce as a measure of family instability given the high cohabitation rates in the countries we analyze.

## Background

### *Why Do Divorce Risks Vary by Parental Education?*

Theorizing about why parental education would matter for their children's union dissolution has been sparse. The existing explanations for the association between parental education and union dissolution can be grouped into those underlining socioeconomic and family demographic pathways and into those theorizing the remaining net association between parental education and union dissolution (e.g., Lyngstad, 2006; Todesco, 2013).

First, parents' education can affect their offspring's family dissolution risks because of the intergenerational transmission of educational attainment. The persistent positive association between parental and offspring's education is among the most consistent findings in the social sciences (Breen & Jonsson, 2005), but whether higher parental education promotes family stability or not through this pathway depends on the relationship between own educational attainment and family dissolution. Higher levels of education were in many countries related to elevated family dissolution risks just a couple of decades ago, but this relationship has today largely disappeared or reversed to a negative one (Goode, 1962; Härkönen & Dronkers, 2006; Matysiak et al., 2014): As family dissolution became more common, it is the lower educated, rather than the higher educated, who experience the highest family dissolution risks. Thus, the role of intergenerational educational transmission in shaping the association between parental education and family dissolution is contingent on the educational gradient of family dissolution that prevails in each society and time period.

Second, parental education can affect the risk of family dissolution through family demographic pathways. Parental separation is a well‐known predictor of individuals' own union dissolution, and this relationship is found in a range of countries (Dronkers & Härkönen, 2008; Wolfinger, 2003). If education was associated with separation risk in the parental generation, then parental separation can be one of the pathways linking parental education to family dissolution. Again, because the educational gradient of separation and divorce varies cross‐nationally and over time, the association between parental education and parental separation can vary as well. Parental separation can thus increase the family dissolution risk among those with highly educated parents or with low educated parents, depending on the association between education and separation in the parental generation.

Parental education is associated with two features of the family formation process that are important predictors of family dissolution, namely, the age at family formation and marriage (e.g., Lyngstad & Jalovaara, 2010). On average, children of higher socioeconomic status (SES) parents form coresidential unions and have children at a later age (e.g., Axinn & Thornton, 1992; Rijken & Liefbroer, 2009; Wiik, 2009), even when their own educational level has been taken into account (Brons, Liefbroer, & Ganzeboom, 2017; Dahlberg, 2015). Later age at family formation is one of the most robust predictors of family stability (Lyngstad & Jalovaara, 2010).

Many studies (e.g., Axinn & Thornton, 1992; South, 2001) have also found that higher parental SES predicts postponement of marriage. However, although those with higher SES parents may marry later, it is less clear whether they are less likely to be married at the time they have children in a coresidential relationship, which are the unions we consider in this study. On one hand, those with higher SES parents have been argued to be less traditional (e.g., Lesthaeghe, 1995). On the other hand, marriage is less reversible than cohabitation, even when children are involved, and higher educated parents and their children generally have a higher stake in the former than in the latter (Wiik, 2009). Furthermore, those from more advantaged backgrounds can be more likely to be married because of their longer partner search and later age at family formation. Recent findings suggest that the association between parental education and the partnership context at entry into parenthood is, too, societally contingent, and low parental education predicts childbearing within cohabitation more strongly in North America and Eastern Europe than in West Europe (Koops, Liefbroer, & Gauthier, 2017).

**Figure 1:**
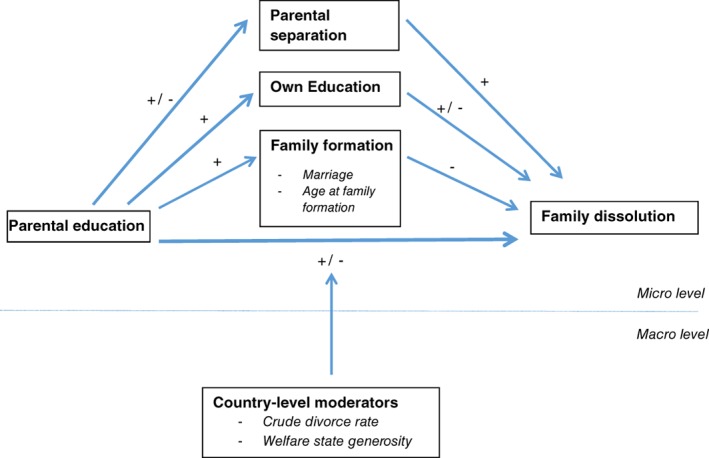
Hypothesized Pathways From Parental Education to Family Dissolution.

Figure 1 summarizes the expected pathways from parents' education to offspring's family dissolution. Although some studies found that the association between parental SES and family dissolution disappeared once observed socio‐economic and demographic factors had been controlled for (Berrington & Diamond, 1999; Bracher et al., 1993; Bumpass et al., 1991; Kiernan, 1986), several studies have reported a remaining, positive relationship (e.g., Hoem & Hoem, 1992; Janssen, 2001; Klijzing, 1992; Lyngstad, 2004, 2006; Todesco, 2013).

This net association has been theorized as reflecting unmeasured class‐related sociocultural factors or financial support from the parents. Hoem and Hoem (1992) speculated that the higher divorce risk of Swedish women from higher class backgrounds reflects these women's and their parents' embrace of a “bourgeois culture,” which is more accepting of divorce (see also Lyngstad, 2004, p. 135, 2006, p. 50). Rijken and Liefbroer (2012) found that education is positively related to approval of divorce among Europeans. Yet higher parental education can also relate to sociocultural factors that stabilize families. In the United States, education has over time become negatively associated with the approval of divorce, which can reflect socioeconomically diverging benefits to stable marriages (Martin & Parashar, 2006). It is also possible that educated parents are more knowledgeable about the (negative) consequences of family dissolution. Although the available evidence is not straightforward (cf. Amato, 1996), the literature on own education and divorce has furthermore argued that educated couples have better interpersonal skills (Blossfeld, de Rose, Hoem, & Rohwer, 1995; Härkönen & Dronkers, 2006), and educated parents can pass these skills on to their children.

Better‐educated parents are in a better situation to financially support their adult children (Lyngstad, 2006; Todesco, 2013). The potential for receiving financial support may lower the threshold for family dissolution by lowering its perceived costs, but parental financial support can alternatively stabilize families faced with economic difficulties (Lyngstad, 2006). Higher socioeconomic background and the economic security it provides while growing up is also related to better mental and physical health (Elo, 2009), which can lower the likelihood of family dissolution (cf. Lyngstad & Jalovaara, 2010). All in all, although previous studies have found no or a positive net association between parental education and family dissolution, there are reasons to expect that the association can—at least in some contexts—be negative as well.

### *Cross‐National and Cohort Variation*

The above discussion has repeatedly suggested that the relationship between parental education and family dissolution is not necessarily constant over time or across societies. Below, we systematize this discussion.

Previously we pointed to the cross‐national and cohort variation in the educational gradient of separation and divorce. This can produce variation in the association between parental education and family dissolution through two pathways. First, variation in the educational gradient of family dissolution in the parental generation means variation in the association between parental education and parental separation. Second, educated parents tend to have educated children. Whether this means that the children of educated parents also have more stable families will vary across societies, depending on the association between own education and family dissolution.

The association between parental education and family dissolution can vary cross‐nationally and over time also because of variation in the relationship between parental education and family formation. According to the second demographic transition theory (e.g., Lesthaeghe, 1995, 2010), nontraditional family forms, such as childbearing within cohabitation, started in the advantaged sections of society, from where it gradually spread to other social groups. However, empirical findings show that the socioeconomic patterns of these changes show important cross‐national variation, and these patterns continue to differ between societies (Koops et al., 2017). Similar cross‐national variation can be found in the link between parental SES and the timing of coresidential unions (Brons et al., 2017).

This leads us to two hypotheses. First, we expect that the gross association between parental education and family dissolution varies cross‐nationally and that this variation diminishes after we control for parental separation, educational attainment, and age and marriage at family formation (Hypothesis 1). Our second hypothesis is more specific and builds on the documented change in the educational gradient of family dissolution (e.g., Härkönen & Dronkers, 2006). We expect that the gross association between parental education and family dissolution has changed from a positive to a negative one because of a changing educational gradient in family dissolution (Hypothesis 2). Note that this change is possible because of changes in the divorce gradient either in the parental or in the filial generations.

We also expect that the net association has changed from positive to negative. Also, this expectation builds on the literature on the changing relationship between individuals' own education and divorce. Goode (1962) theorized that divorce was the privilege of the privileged in societies where divorcing was difficult and required resources for dealing with its legal and social consequences, but as divorcing became easier and more common it became accessible to the lower socioeconomic strata as well. If the perceived benefits of stable family life have diverged, those with higher education may have developed more restrictive attitudes toward it (Martin & Parashar, 2006). These mechanisms can extend beyond the association between achieved status (own education) and family dissolution to that between ascribed status (parental education) and family dissolution (cf. Todesco, 2013). Moreover, traits that stabilize families may have become more important in high divorce contexts, and to the extent that parental education promotes such traits, it will increasingly promote family stability. Therefore, we expect that the net association between parental education and family dissolution has changed from positive to negative (Hypothesis 3).

The previous discussion suggests that the net association between parental education and family dissolution is different in societies with a high divorce rate compared to a low one. The related empirical literature on own education and divorce has similarly reported that the educational gradient of divorce tends to be (more) negative in times and societies in which it is more common to divorce (Härkönen & Dronkers, 2006; Matysiak et al., 2014). Our theorizing of the net association between parental education and family dissolution suggests similar patterns, and we thus expect that the net association between parental education and family dissolution is positive in societies with low divorce rates, but nil or even negative in societies with high divorce rates (Hypothesis 4).

Last, we discussed how the net association between parental education and family dissolution can reflect differences in financial conditions and economic support from the parents, although whether this would (de)stabilize families is not obvious. Parents' financial resources can lower the threshold of family dissolution by providing (the promise of) means to deal with its consequences. On the other hand, these means can stabilize families by lowering financial stress or foster traits during childhood that enhance family stability. Either way, parental financial resources should play a smaller role in welfare states that are more generous. Härkönen and Dronkers (2006) found that the educational gradient of divorce was less negative in such contexts. We hypothesize that the net association between parental education and family dissolution is weaker in countries with a generous welfare state (Hypothesis 5).

## Method

### *Data*

We use data from 17 European countries. Data for 16 countries come from the first wave of the GGS. The data were collected in different years in the different countries, between 2002 and 2013 (Fokkema, Kveder, Hiekel, Emery, & Liefbroer, 2016). We chose the countries for which sufficiently detailed information was available on the partnership history, parental and individual educational attainment, and parental separation, namely, Austria, Belgium, Bulgaria, Czech Republic, Estonia, France, Georgia, Hungary, Italy, Lithuania, Netherlands, Norway, Poland, Romania, Russia, and Sweden. For the United Kingdom, we use the Harmonized Histories data set created by the Non‐Marital Childbearing network and made publicly available to the Gender and Generations Programme research community (for information, see Perelli‐Harris, Kreyenfeld, & Kubisch, 2010). The Harmonized Histories data set consists of data from the British Household Panel Survey, collected in 2005 and 2006 and made comparable to GGS. Missing or inconsistent data led us to exclude some interesting countries. The U.S. data in the Harmonized Histories data set does not have information on parental separation, and this variable is not correct in the German GGS. The Australian GGS does not include information on unmarried cohabiting couples.

We excluded the oldest childbearing union cohorts, which started before 1970, because our country‐level indicators (discussed later) are not representative for them (available from 1970 onward). Moreover, we excluded all respondents without children because we focus on the dissolution of childbearing unions, which resulted in a sample of 92,862 respondents. Furthermore, we excluded respondents with missing information on at least one of the independent variables, leading to our analytical sample of 84,045 men and women in 17 countries who had their first child within a coresidential union after 1970. The overall percentage of respondents with missing variables was 9.5%, ranging from 2% in Italy to 22% in Russia. Parental and own education and parental separation were the variables with most missing information. As a robustness check, we performed our analyses with multiple imputed data and found that the results were almost identical to the ones with our analytical sample.

### *Dependent Variable*

Our dependent variable is family dissolution, defined as the dissolution of one's first childbearing union (irrespective of marital status). Most previous studies have focused on divorce, but because of the increase in cohabitation as a stable family form (Andersson, Thomson, & Duntava, 2017; Heuveline & Timberlake, 2004), focusing on divorce is too restrictive, especially in light of the cross‐national and cohort coverage of our data. Childbearing unions, that is, coresidential unions involving a common child, are a more comparable family type as they can in all European countries be seen as a stable and serious relationship form.

Of the 84,045 respondents in our analytical sample, 15,774 (19%) dissolved their childbearing union within the observation window. We converted the data into a person‐year format for discrete‐time event history analyses (Allison, 1984), which we chose because of the ease of handling time‐varying covariates (in our case, individuals' own educational attainment). The results are robust to using months as the units of analysis or Cox regression as the method. The respondents become at risk of family dissolution when their first child was born within a coresidential union, irrespective of marital status. They were followed until the year of the separation, until the year of the interview, or up to a maximum of 20 years.

### *Independent Variables*

Our main independent variable is parental education. The highest level of education of both parents is available for all 17 countries, which we converted into a comparative measure of educational level, the International Standard Level of Education (ISLED; Schröder & Ganzeboom, 2014). Its advantage over the International Standard Classification of Education (ISCED) is that the ISLED is more fine grained, is sensitive to differences in educational systems between countries, and allows for continuous scaling (range 0–100). We use the average score of fathers' and mothers' education because we are interested in the overall effect of parental education rather than whether fathers or mothers are more influential. This average score was standardized to a Z‐metric (mean = 0, standard deviation = 1) within each country. The results were robust to using the highest parental level of education instead of the average.

Parental separation is measured by a dummy variable, which is unity if the parents ever separated and zero if not. Time‐varying information on the respondents' highest level of completed education was also converted into ISLED and expressed in a Z‐standardized metric within each country. The last two mediating variables are the age at the start of the childbearing union, which ranges from age 15 to age 60, and a dummy variable indicating whether the respondent was married at the beginning of the union. We control for gender in each model. Union duration is expressed as linear and squared years since the beginning of the union. The year in which the childbearing union started (union cohort) was used to construct a continuous cohort variable (cohorts ranged between 1970 and 2013). The country‐specific descriptives of all independent variables and of the dependent variable can be found in Table 1.

**Table 1:** Descriptive Statistics for the Dependent and Independent Variables, Separately for Each Country

	*N*	% dissolution	% women	Mean union r	Mean duration	Mean parental education (ISLED: 0–100)	% experienced parental separation	Mean own education (ISLED: 0–100)	Mean age childbearing union	% married when union started	Average crude divorce rate^a^	Average crude divorce rate^b^	Welfare generosity ^a^ (social transfers as % of GDP)	Welfare generosity b (social transfers as % of GDP)
All	84,045	18.77	56.58	1987.72	13.07	37.92	12.77	54.24	26.07	89.58	1.84	2.31	15.04	13.91
Austria	2,493	18.53	66.23	1997.59	8.95	50.13	18.69	64.38	26.32	80.67	1.72	2.25	16.51	18.68
Belgium	3,361	19.90	53.91	1989.94	12.99	40.66	12.14	56.67	27.52	87.86	1.27	2.97	16.06	15.72
Bulgaria	6,028	9.74	59.92	1987.50	13.27	35.01	8.68	47.60	24.02	91.49	1.39	1.52	–	10.51
Czech Rep.	3,760	24.15	55.13	1986.44	13.03	45.87	14.76	53.07	25.11	94.47	2.57	2.92	–	12.15
Estonia	3,675	29.82	63.48	1985.67	12.39	41.12	21.88	55.52	24.61	83.10	3.58	3.50	–	10.00
France	4,296	22.81	55.59	1987.61	12.26	32.35	13.64	49.50	27.02	80.80	1.37	2.22	16.03	17.58
Georgia	4,866	8.28	60.30	1987.26	14.47	43.46	4.01	54.65	25.15	86.56	1.12	0.75	–	–
Hungary	5,883	20.26	56.33	1985.17	13.45	37.39	12.68	50.96	24.85	94.63	2.48	2.40	–	14.16
Italy	4,661	8.77	53.12	1986.15	13.49	22.72	2.27	43.92	28.46	98.28	0.30	0.73	14.40	16.53
Lithuania	4,132	19.97	50.19	1987.62	13.39	38.25	11.16	55.75	25.62	96.42	2.72	3.13	–	10.92
Netherlands	3,731	19.46	60.44	1987.17	11.81	38.62	10.10	56.47	28.62	90.83	1.57	2.06	17.02	12.88
Norway	7,397	22.77	51.82	1988.29	12.73	34.85	13.46	57.61	27.21	80.21	1.43	2.29	12.64	14.22
Poland	10,490	14.60	58.14	1988.29	14.43	38.85	7.43	57.87	25.47	96.42	1.19	1.49	–	15.58
Romania	5,882	11.14	50.39	1985.95	14.46	28.87	18.04	44.52	25.40	96.43	0.91	1.59	–	10.03
Russia	4,679	28.87	63.11	1985.97	12.26	41.88	17.74	60.97	24.10	93.14	3.53	4.47	–	–
Sweden	4,969	25.84	52.81	1990.86	12.78	44.08	22.78	59.09	28.36	73.90	1.98	2.42	15.99	16.54
United Kingdom	3,742	27.02	57.70	1988.24	11.30	43.23	19.00	58.43	27.26	86.91	2.10	2.53	11.62	13.14

*Note*. GDP = gross domestic product; ISLED = International Standard Level of Education.

a Average for the oldest union cohort (1970–1987).

b Average for the youngest union cohort (1988–2013).

### *Country‐Level Indicators*

We use country‐level measures of the crude divorce rate and welfare state generosity, which we use to assess whether the association between parental education and family dissolution is modified by these country‐level characteristics (Hypotheses 4 and 5). In both cases, we constructed separate measures for an old cohort (start of childbearing union before 1988) and a young cohort (1988 or later) to account for changes in the divorce rate and welfare states. The cutoff point of 1988 divides the number of respondents evenly between the two cohorts and ensures a sufficient number of respondents as well as non missing values for the macro‐level variables for each country cohort.

The average crude divorce rate per country for the older cohort is the average of crude divorce rates from the years 1970 and 1985, derived from World Marriage Data 2008 (United Nations, 2009). The average divorce rate for the youngest cohort is based on the country‐specific crude divorce rates for 1995 and 2005 derived from *World Marriage Data 2008* (United Nations, 2009) and for the year 2011 derived from the *United Nations Demographic Yearbook (2013)* (United Nations, 2013). Although not a perfect measure of family instability, the crude divorce rate is a readily available aggregate measure that correlates highly with more appropriate measures (Amato, 2010). It thus serves as a proxy for costs and availability of family dissolution.

To test whether welfare state generosity modifies the cross‐national variation in the net association between parental education and family dissolution (Hypothesis 5), we calculated for each country cohort the average social security transfers as a share of the gross domestic product, derived from the Comparative Political Data Set (Armingeon, Isler, Knöpfel, Weisstanner, & Engler, 2016). Unfortunately, there were no data available for Russia and Georgia, and only for half of the countries for the old cohort. Because of this, the analysis using welfare state generosity is based on a more restricted sample of 23 country cohorts. The descriptives of these macro‐level indicators can also be found in Table 1.

### *Analytical Strategy*

Our analysis proceeds in three steps. First, we estimate discrete‐time event history regressions separately for each country. We estimate two models. The baseline model estimates the gross association between parental education and family dissolution, controlling for gender, duration (linear and squared), and year of birth. The second model estimates the net association between parental education and family dissolution after adding the mediators parental separation, individuals' own educational attainment, the age at the start of the childbearing union, and whether the couple was married at the beginning of the union. In additional analyses, commented on in the text and presented in the Appendix, we entered these mediators stepwise (first, the parental separation, then educational attainment and finally, the family formation variables). The results were almost identical to those estimated with the Karlson–Holm–Breen (Karlson, Holm, & Breen, 2012) method, which is immune to the rescaling bias in logistic regression models, so we present the more familiar odds ratios from the discrete‐time event history models. The results were also robust when we analyzed multiple imputed data, as mentioned previously.

We summarize the cross‐national variation in these estimates by using tools generally employed in meta‐analyses. We did this because of the small number of countries in our study (*N* < 30), which restricts the use of multilevel models (Bryan & Jenkins, 2016). Using the estimated odds ratios and their confidence intervals as input, we estimate the between‐country heterogeneity coefficient I^2^, which is the percentage of observed total variation across countries as a result of real heterogeneity rather than chance. I^2^ is calculated as 100% × (*Q − df)/Q*, where *Q* is Cochran's heterogeneity statistic and *df* stands for degrees of freedom (Harris et al., 2008). I^2^ ranges between 0% and 100%. Estimates above 50% can be interpreted as indicating “substantial” cross‐national variation, and estimates above 75% indicate “considerable” cross‐national variation (cf. Higgins, Thompson, Deeks, & Altman, 2003). I^2^ for the estimates from the first model tells about cross‐national variation in the gross association between parental education and family dissolution risk, and I^2^ for the second model tells about the cross‐national variation in the net association. I^2^ was estimated using the metan command in Stata 14 (StataCorp, 2015).

Second, we analyze whether the gross and net associations have changed over time. We estimate the baseline models with an interaction term between union cohort and parental education, separately for each country. We continue to analyze the countries in which the gross association has changed, selected using likelihood ratio tests. We first add the four mediating variables to assess whether these family demographic and socioeconomic pathways explain any of the change in the association between parental education and family dissolution. Then we add an interaction term between union cohort and own education to assess whether changes in the educational gradient of family dissolution explain changes in the parental educational gradient.

Third, we analyze whether the net associations—the estimates from the second discrete‐time event history model—are systematically associated with our contextual variables. Again, as a result of our small number of countries, multilevel models would not be appropriate in particular for estimating of the cross‐level interactions between the parental education and the time‐varying, country‐level variables (Bryan & Jenkins, 2016). Using meta‐analytic tools (meta‐regressions) for cross‐national data, the country‐specific estimates of the net association of parental education and family dissolution are regressed separately on the contextual variables (Harbord & Higgins, 2008). Because the country‐level indicators changed over time, we divided our sample into two groups (an old [before 1988] and young union cohort [1988 and later]) and regressed the available country‐cohort‐specific estimates of the net effect of parental education on the macro‐level indicators. The samples for the respective analyses are the 34 country cohorts for which we had information on the crude divorce rate and the 23 country cohorts for which we had measures of welfare state generosity. The country cohorts are weighted by the inverse of the standard error so that those with more precise estimates have more influence. These models were estimated using the robumeta command in Stata 14 (StataCorp, 2015), because with this command we could cluster estimates by country.

## Results

### *Parental Education and Dissolution in 17 Countries*

**Figure 2:**
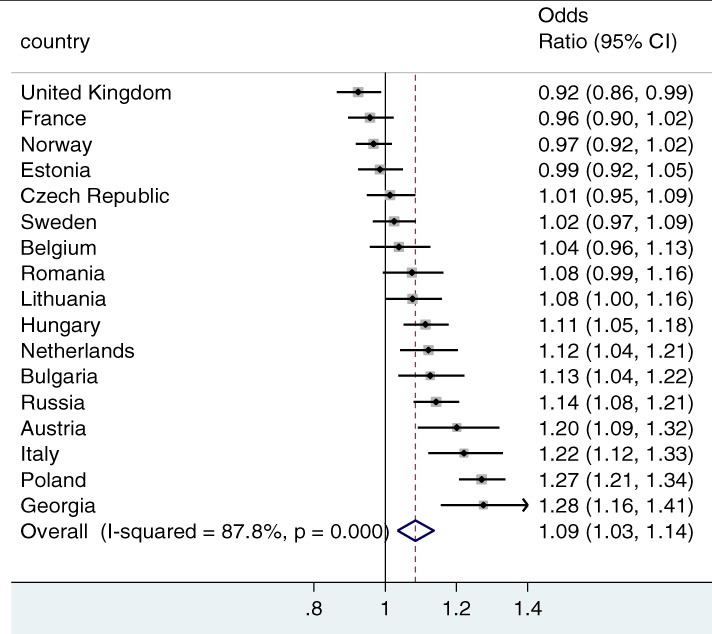
The Gross Association Between Parental Education and Family Dissolution. *Note*. Meta‐analysis with discrete‐time event history models (odds ratios and 95% confidence intervals are presented). Controlled for gender, year childbearing union started, duration, and duration squared.

**Figure 3:**
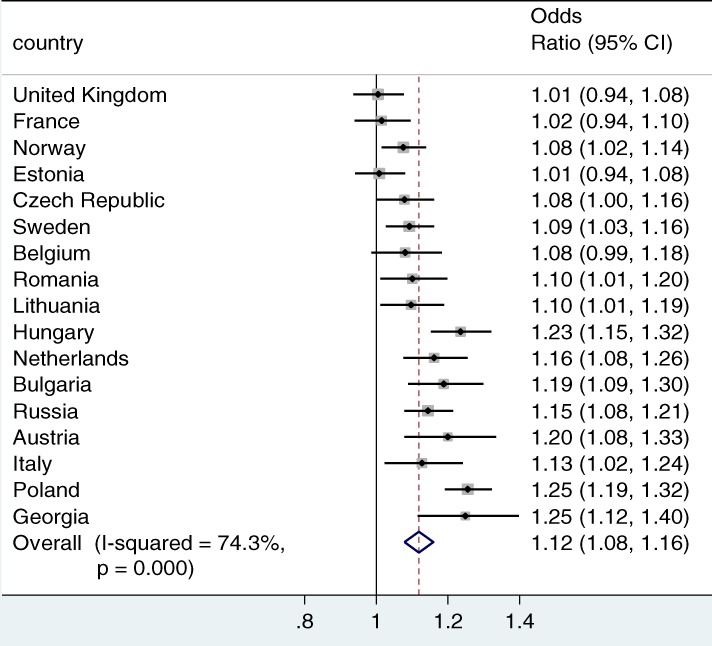
The Net Association Between Parental Education and Family Dissolution. *Note*. Meta‐analysis with discrete‐time event history models (odds ratios and 95% confidence intervals are presented). Controlled for gender, year childbearing union started, duration, duration squared, parental separation, own education, age at family formation, and married at family formation.

Figure 2 shows the gross associations between parental education on union dissolution (the baseline model), and Figure 3 shows the net associations (thus, after adjusting for the mediating variables), respectively, for each country. The figures present the point estimates and the 95% confidence intervals for the odds ratios. The diamond at the bottom of the figures presents the average estimate for the 17 countries, inversely weighted by their standard errors to take into account the precision of the estimates. The I^2^ provides an estimate of the cross‐country heterogeneity in the associations.

The countries were ordered by the ascending gross association. In the United Kingdom, having highly educated parents was associated with a lower risk of family dissolution (Figure 2). The association was not statistically significant in six countries (France, Norway, Estonia, the Czech Republic, Sweden, and Belgium), and positive in the remaining 10 countries. In these countries, having highly educated parents was associated with a higher risk of family dissolution. The positive association was the strongest in Italy, Poland, and Georgia. The overall effect was likewise positive (odds ratio = 1.09), but the I^2^ estimate of 87.8% confirmed the considerable cross‐country variation in these associations.

Figure 3 presents the net associations between parental education and family dissolution risk. The stepwise models are found in the Appendix (Figures A1 and A2). Figure 3 shows that the overall association between parental education and family dissolution became marginally more positive after including parental separation, individuals' own education, and age and marriage at the start of the childbearing union as mediators (odds ratio = 1.12). The cross‐national heterogeneity decreased somewhat (I^2^ = 74.3%), although it could still be interpreted as being considerable.

With the clearest exception of Italy, the estimates moved toward more positive ones in each country. This result was confirmed with the Karlson–Holm–Breen method, which is not sensitive to rescaling unlike nonlinear methods. After adjusting for the mediating variables, no negative and statistically significant associations remained. The associations were not statistically significant in the United Kingdom, France, Estonia, and Belgium, and positive and significant in all the other countries. The shift toward more positive associations was the clearest in Norway and Czech Republic, where the associations changed from no association to a positive association.

The difference between the gross and net effects generally implied that higher parental education was in many countries associated with pathways that promote family stability, which also suppressed the mostly positive net association between parental education and family dissolution. The stepwise analyses, shown in the Appendix, pointed to own educational attainment and family formation as such important pathways. Regarding the former, highly educated parents tended to have highly educated children, who in many of the countries were less likely to experience family dissolution. This pattern was the opposite in Italy, where own education was positively associated with family dissolution. Children of highly educated parents were also often older and more likely to be married at the beginning of their childbearing union, which stabilized their families.

### *Did the Parental Educational Gradient Change?*

We hypothesized that the gross as well as the net associations between parental education and family dissolution changed from positive to negative (Hypotheses 2 and 3). To test these hypotheses, we first ran interaction models separately for each country, interacting parental education with the year in which the childbearing union started (union cohort), with controls for gender, duration, and duration squared. This model tested whether there had been a shift in the gross association between parental education and family dissolution.

This interaction model improved the model fit at the 5% level of significance (assessed by likelihood ratio tests) in five countries (Belgium, Bulgaria, Norway, Sweden, and the United Kingdom). In addition, the interaction was significant in Austria once we controlled for the mediating variables (Model 2). These findings imply that the gross or net (or both) association between parental education and family dissolution had changed over time in these six countries, which we focus more closely on next.

Table 2 presents the results from three models. The union cohorts were centered at the mean union cohort for each country, and the estimate for parental education showed that the gross association between parental education and family dissolution risk was, in those cohorts, positive in Austria and Bulgaria; zero in Belgium, Norway, and Sweden; and negative in the United Kingdom. The interaction coefficient indicated that the association became (more) negative over time in all countries but Austria, where the coefficient was not significant. Furthermore, predictions based on the model showed that the gross association switched from positive to negative in Belgium, Bulgaria, Norway, and Sweden during the observation window, and a negative association opened up in the United Kingdom (not shown).

**Table 2:** Discrete‐Time Event History Analysis of Cohort Change in the Association Between Parental Education and Family Dissolution

Countries	Variables	Model 1	Model 2	Model 3
Austria	Parental education	1.144 (0.064)*	1.112 (0.066)	1.109 (0.068)
*N* = 2,493	Year union started	0.983 (0.009)*	0.980 (0.010)	0.981 (0.010)
	**Parental Education × Union Year**	**0.986 (0.008)**	**0.977 (0.008)** **	**0.976 (0.009)** **
	Parental separation		1.682 (0.183)**	1.683 (0.183)**
	Own education		0.969 (0.045)	0.977 (0.068)
	Age at family formation		0.950 (0.013)**	0.950 (0.013)**
	Married		0.212 (0.023)**	0.212 (0.023)**
	Own Education **×** Union Year			1.001 (0.006)
	Likelihood‐ratio χ^2^ (*df*)	36.46 (6)	293.37 (10)	293.39 (11)
Belgium	Parental education	1.021 (0.042)	1.061 (0.049)	1.059 (0.049)
*N* = 3,361	Year union started	1.020 (0.005)**	1.017 (0.005)**	1.017 (0.005)**
	**Parental Education × Union Year**	**0.988 (0.004)** **	**0.987 (0.004)** **	**0.988 (0.004)** **
	Parental separation		1.448 (0.164)**	1.450 (0.164)**
	Own education		0.965 (0.041)	0.968 (0.039)
	Age at family formation		0.950 (0.010)**	0.950 (0.009)**
	Married		0.401 (0.051)**	0.401 (0.051)**
	Own Education **×** Union Year			0.998 (0.003)
	Likelihood‐ratio χ^2^ (*df*)	65.81 (6)	162.80 (10)	163.04 (11)
Bulgaria	Parental education	1.098 (0.047)*	1.163 (0.054)**	1.160 (0.054)**
*N* = 6,028	Year union started	1.011 (0.006)	1.001 (0.006)	1.001 (0.006)
	**Parental Education × Union Year**	**0.985 (0.005)** **	**0.988 (0.005)** *	**0.987 (0.005)** *
	Parental separation		1.808 (0.226)**	1.814 (0.227)**
	Own education		0.981 (0.039)	0.988 (0.043)
	Age at family formation		0.988 (0.010)	0.988 (0.010)
	Married		0.439 (0.064)**	0.436 (0.064)**
	Own Education **×** Union Year			1.002 (0.004)
	Likelihood‐ratio χ^2^ (*df*)	41.94 (6)	98.77 (10)	99.01 (11)
Norway	Parental education	0.952 (0.026)	1.058 (0.032)	1.061 (0.032)*
*N* = 7,397	Year union started	1.013 (0.003)**	0.992 (0.003)*	0.992 (0.003)*
	**Parental Education × Union Year**	**0.989 (0.003)** **	**0.991 (0.003)** **	**0.993 (0.003)** *
	Parental separation		1.517 (0.102)**	1.515 (0.102)**
	Own education		0.914 (0.025)**	0.907 (0.026)**
	Age at family formation		0.953 (0.006)**	0.953 (0.006)**
	Married		0.234 (0.015)**	0.235 (0.015)**
	Own Education **×** Union Year			0.997 (0.003)
	Likelihood‐ratio χ^2^ (*df*)	62.41 (6)	747.07 (10)	748.29 (11)
Sweden	Parental education	0.998 (0.031)	1.065 (0.034)*	1.069 (0.034)*
*N* = 4,969	Year union started	1.003 (0.003)	0.996 (0.003)	0.995 (0.003)
	**Parental Education × Union Year**	**0.987 (0.003)** **	**0.987 (0.003)** **	**0.989 (0.003)** **
	Parental separation		1.543 (0.100)**	1.550 (0.101)**
	Own education		0.943 (0.029)	0.896 (0.027)**
	Age at family formation		0.938 (0.006)**	0.939 (0.027)**
	Married		0.276 (0.017)**	0.276 (0.017)**
	Own Education **×** Union Year			0.992 (0.002)**
	Likelihood‐ratio χ^2^ (*df*)	48.75 (6)	661.37 (10)	673.31 (11)
United	Parental education	0.909 (0.032)**	0.989 (0.036)	0.989 (0.036)
Kingdom	Year union started	1.025 (0.004)**	1.019 (0.004)**	1.019 (0.004)**
*N* = 3,742	**Parental Education × Union Year**	**0.992 (0.004)** *	**0.993 (0.004)** *	**0.993 (0.004)#**
	Parental separation		1.211 (0.095)*	1.211 (0.095)*
	Own education		0.884 (0.031)**	0.885 (0.038)**
	Age at family formation		0.929 (0.007)**	0.929 (0.007)**
	Married		0.322 (0.032)**	0.322 (0.032)**
	Own Education **×** Union Year			1.000 (0.004)
	Likelihood‐ratio χ^2^ (*df*)	111.74 (6)	455.84 (10)	455.85 (11)

*Note*. Odds ratios and standard errors are presented. All models are additionally controlled for gender, duration, and duration squared.

# *p* < .10.

* *p* < .05.

** *p* < .01.

The bold data show the cohort change in the parental educational gradient, which is the main result of this table.

The second model added controls for parental separation, own education, age at family formation, and marriage at family formation. Having separated parents increased the family dissolution risk, whereas being older and married at the beginning of the childbearing union had stabilizing effects. Own education was negatively associated with family dissolution risk in Norway, Sweden, and the United Kingdom, but there was no association in the other three countries. More important for this study, the interaction effect between union cohort and parental education remained almost unchanged in Belgium, Sweden, and the United Kingdom, but became smaller in Bulgaria and Norway, and maybe surprisingly, larger and significant in Austria.

The third model included the interaction between own education and union cohort. Model 3 showed that in all countries, there remained a significant change in the association between parental education and family dissolution, also after including the changing educational gradient of family dissolution in the children's generation. The interaction between own education and union cohort was significant and negative only in Sweden. In Sweden, the interaction between parental education and birth cohort became smaller, but remained significant. Thus, in Sweden, the increasingly negative educational gradient of family dissolution in the children's generation had been partly responsible for the changing association between parental education and family dissolution.

### *Variation by divorce rate and welfare state generosity*

**Figure 4:**
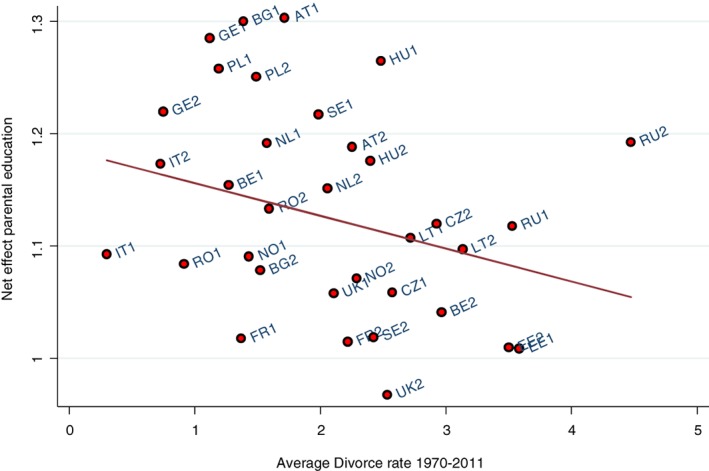
The Association Between the Net Effect of Parental Education and Union Dissolution, and the Average Crude Divorce Rate. *Note*. 1 = old union cohort (1970–1987); 2 = young union cohort (1988–2013). *b* = −0.029; *p* = .283.

**Figure 5:**
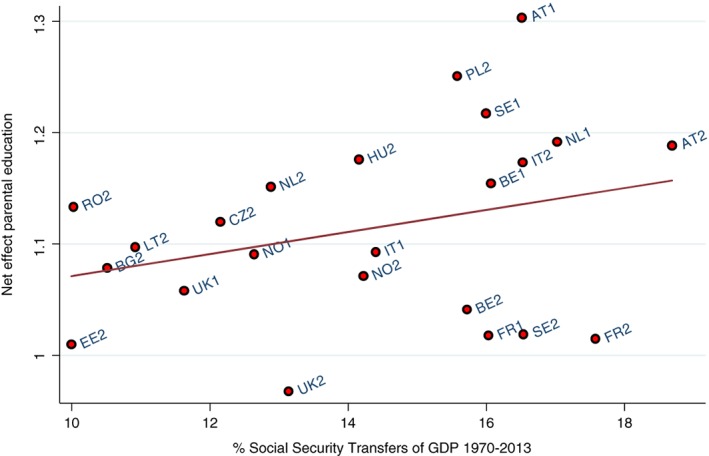
The Association Between the Net Effect of Parental Education on Union Dissolution and Average Social Security Transfers (as % of Gross Domestic Product). *Note*. Social Security Transfer data not available for Russia and Georgia. 1 = old union cohort (1970–1987); 2 = young union cohort (1988–2013). *b* = 0.010; *p* = .245.

Figures 4 and 5 present the results of the analysis of the moderating role of the crude divorce rate and welfare state generosity on the net association of parental education and family dissolution. To account for changes in the net association as well as the crude divorce rate and welfare state generosity, we divided our sample into an old and young cohort. The net association within each country cohort was regressed on these country‐level indicators.

We expected to find a negative association between the average crude divorce rate and the parental educational gradient of family dissolution (Hypothesis 4). Figure 4 indeed shows that the net association between parental education and family dissolution risk tended to be clearly positive in country cohorts where the average divorce rate was low, but weak in country cohorts where the average divorce rate was high. Despite the negative slope (*b* = −0.029), the association was not statistically significant. When we excluded the younger Russian cohort as an influential outlier—identified as such by its crude divorce rate that was more than 1.5 times interquartile range after the third quartile (Tukey, 1977)—the association became stronger and statistically significant (*b* = −0.053, *p* = .03; see Figure A3 in the Appendix). Thus, there was some evidence of a negative association between the divorce rate and the net association.

We also expected that the net association between parental education and family dissolution was stronger in countries with a less generous welfare state than in countries with a more generous welfare state (Hypothesis 5). Figure 5 shows no clear pattern between the net association of parental education on family dissolution and welfare state generosity (*b* = 0.010, *p* = nonsignificant). Unlike in Figure 4, there were no influential outliers that affected the results.

## Discussion

We analyzed the association between parental education and family dissolution in 17 European countries. The scholarly attention to educational differences in family demography and to family background effects on life chances has not translated to similar interest in parental education and family stability, and our study is the first cross‐national analysis on this subject. Documenting the parental background differences in family stability contributes to a more comprehensive understanding of stratification in family demography, and analysis of its cross‐national differences adds to the understanding of the societal factors associated with this stratification.

In most countries, having highly educated parents is either not related to the risk of family dissolution or it predicts a higher dissolution risk. This was true (with one exception, the United Kingdom) for the gross association, and the view of a positive association was reinforced once adjusting for parental separation, own educational attainment, and age and marital status at family formation. Our findings of a generally positive association are mostly in line with earlier ones (cf. Lyngstad & Jalovaara, 2010), but systematize these results by being the first cross‐national analysis of parental educational differences in family dissolution.

Both the gross and the net associations between parental education and family dissolution showed considerable cross‐national variation, although the variation in the association diminished, as expected, once we adjusted for some important socioeconomic and family demographic pathways (in support of our first hypothesis). However, partly contrasting our second and third hypotheses, we found general stability in the gross and net associations. The gross association had changed (toward more negative) in five of the 17 countries, and the net association showed similar change in six of the 17 countries. In general, the change in the association between parental education and family dissolution in these countries could not be explained by sociodemographic factors or by the changing association of own education and family dissolution. In the remaining 11 countries, the parental educational gradient of separation has remained stable. In addition to differences in sample sizes and the cohorts covered, the countries where the gross or net association changed can have been more advanced in the second demographic transition (the United Kingdom, Norway, and Sweden as the best examples), but strong conclusions are difficult to draw.

To better understand the variation across countries and over time, we divided the data into an old and young cohort in each country and used techniques familiar from meta‐analysis to regress the net association between parental education and family dissolution in these country cohorts on the crude divorce rate and on welfare state generosity. We hypothesized that the net association should be positive in country cohorts with a lower divorce rate (Hypothesis 4) and in country cohorts with a less generous welfare state (Hypothesis 5), but nil or even negative when the divorce rate is higher and the welfare state more generous. We found suggestive evidence for the fourth hypothesis, which was stronger after excluding Russia as an influential case from the analysis. Although similar to other Eastern European countries with regard to many features of the second demographic transition (Lesthaeghe, 2010), Russia has a higher divorce rate that set it as an outlier in the analysis. Possible reasons range from a high prevalence of unintended pregnancies and subsequent “shotgun” marriages (Zakharov, 2008) to the social turmoil that followed the decline and collapse of the Soviet Union. In contrast to some support for the fourth hypothesis, we did not find that the generosity of the welfare state modified the net association between parental education and family dissolution. However, we have to keep in mind that the country‐level measures are averages over long periods (around 20 years); although averaging reduces measurement error because of short‐term fluctuations, our long‐term averages can hide trends that shape the parental education–family dissolution relationship. We chose this conservative strategy in response to criticisms of the use of multilevel modeling—which can include cross‐classified random effects of the country level and time—with a limited number of countries (Bryan & Jenkins, 2016). Nevertheless, it is possible that we may have erred on the conservative side in analyzing the country‐level moderators.

Lacking direct measures, the cross‐national analysis provides indirect evidence for the hypothesized mechanisms behind the net relationship between parental education and family dissolution. Despite a general lack of theorizing of this association, the suggested mechanisms can be grouped into sociocultural mechanisms that emphasize class differences in (the intergenerational transmission of) divorce‐friendly values and outlooks and into economic mechanisms that underline the financial support better‐educated parents can provide their children. Related to the latter, we expected that family dissolution would be more strongly associated with parental education in less generous welfare states (our fifth hypothesis). This was not the case. Our suggestive finding that the net association was related to the crude divorce rate is more in line with the sociocultural explanation. When divorcing means breaking social and legal norms, it requires social and cultural resources that often come with high (ascribed or attained) status, but these resources become less important when divorce is democratized (Goode, 1962). Previous research has found evidence for this interpretation regarding the educational gradient of divorce (Härkönen & Dronkers, 2006; Matysiak et al., 2014), and our study extended this to the association between parental education and family dissolution.

Research on the stratification of family dissolution has documented large variation in the relationship between own educational attainment and family dissolution over time and across countries (Härkönen & Dronkers, 2006; Martin, 2006; Matysiak et al., 2014). We found variation in the relationship between parental education and family dissolution as well, but this variation appears less dramatic than the one between own education and family dissolution. Although the size of the relationship between parental education and family dissolution varies considerably, it is generally positive—this is especially clear in the case of the net association—whereas the educational gradient of family dissolution has more clearly varied both in size and in sign (Blossfeld et al., 1995; Härkönen & Dronkers, 2006; Matysiak et al., 2014). Similarly, the educational gradient of family dissolution has changed in several countries, often from positive to negative (Chan & Halpin, 2008; De Graaf & Kalmijn, 2006; Härkönen & Dronkers, 2006; Hoem, 1997; Raymo & Iwasawa, 2017), whereas our findings point primarily to stability in the relationship between parental education and family dissolution.

These results add nuance to perspectives on stratification in family dissolution, which is dominated by views of the increasingly lower status nature of family instability (e.g., McLanahan, 2004). Parental and individuals' own education are of course not the same, but our findings suggest that family dissolution is not generally and increasingly related to low social status regardless of the status measure. Instead, the generally positive association between parental education and family dissolution suggests first of all that family background may ameliorate the inequality consequences of family instability. Second, our findings also show that high parental background does not always lead to positive outcomes with regard to family demography and future life chances, given that the association between parental education and family dissolution is positive and the dominantly negative consequences of family dissolution on adults and children.

This finding also raises intriguing questions about the impact of parental and own education on family dissolution because it is far from obvious to expect that parental and own education predict family dissolution in opposite ways, as seems to be the case in many countries (see also Lyngstad, 2006; Lyngstad & Jalovaara, 2010). Future research should formulate additional hypotheses about why parental and own education can predict family dissolution in opposite ways. Future research should also assess whether our conclusion of relative stability in the association between parental education and family dissolution holds; it is possible that is has become mostly apparent in more recent cohorts and thus not discovered by our linear trend analysis over many cohorts or that there has been change in countries we did not analyze. Understanding these questions would contribute to understanding stratification in family dissolution more broadly.

Family dissolution is socially stratified. Our analysis has contributed to understanding this stratification by showing how parental education predicts family dissolution in different countries and over time. A key lesson is that differences in family instability by ascribed status can be quite different from that by achieved status.

## Note

The authors would like to thank the participants in various conferences and seminars for valuable comments. We also thank Stockholm University Demography Unit for hosting the research visit of Anne Brons. The research leading to these results has received funding from the European Research Council under the European Union's Seventh Framework Programme (FP/2007‐2013)/European Research Council Grant Agreement 324178 (Project: Contexts of Opportunity. Principle Investigator: Aart C. Liefbroer), and from the Strategic Research Council of the Academy of Finland (Decision Number 293103) for the research consortium Tackling Inequality in Time of Austerity.

## Supporting information


**Figure A1** The association between parental education and family dissolution, controlled for parental separation. Meta‐analysis with discrete‐time event history models for 17 European countries (odds ratios and 95% confidence intervals are presented).
**Figure A2** The association between parental education and family dissolution, controlled for parental separation and own education. Meta‐analysis with discrete‐time event history models for 17 European countries (odds ratios and 95% confidence intervals are presented).
**Figure A3** The association between the net effect of parental education and union dissolution, and the average crude divorce rate (without Russia as influential case). b = −0.053; p = .033.
