# Supplementary material for: Parental Education and Family Dissolution: A Cross‐National and Cohort Comparison
Source: J Marriage Fam. 2018 Jan 30;80(2):426–43. doi: 10.1111/jomf.12461 (PMC5888197; doi:10.1111/jomf.12461)
Supplement: Supplementary file 1 — Figure A1 The association between parental education and family dissolution, controlled for parental separation. Meta‐analysis with discrete‐time event history models for 17 European countries (odds ratios and 95% confidence intervals are presented). Figure A2 The association between parental education and family dissolution, controlled for parental separation and own education. Meta‐analysis with discrete‐time event history models for 17 European countries (odds ratios and 95% confidence intervals are presented). Figure A3 The association between the net effect of parental education and union dissolution, and the average crude divorce rate (without Russia as influential case). b = −0.053; p = .033. [file JOMF-80-426-s001.docx]

**Parental education and family dissolution: A cross-national and cohort comparison**

*Online Supporting Materials*

*M.D. (Anne) Brons (corresponding author) ^1,2^*

*J. Härkönen ^3^*

^1^ Netherlands Interdisciplinary Demographic Institute, The Hague (NIDI/KNAW), University of Groningen

^2^ Department of Sociology, VU University Amsterdam, The Netherlands

^3^ Department of Sociology, Stockholm University, Sweden

This document contains the Online Supporting Materials (Appendix) referred to in the main text and includes three Figures that are relevant to our study, but could not be accommodated in our main text.

**Content**

1. Figure A1 which consists of the association between parental education and family dissolution, controlled for parental separation.
2. Figure A2 which consists of the association between parental education and family dissolution, controlled for parental separation and individuals’ own education.
3. Figure A3 which consists of the association between the net effect of parental education and union dissolution, and the average crude divorce rate, without Russia as influential case.

| Figure A1. The association between parental education and family dissolution, controlled for parental separation. Meta-analysis with discrete-time event history models for 17 European countries (odds ratios and 95% confidence intervals are presented). | Figure A2. The association between parental education and family dissolution, controlled for parental separation and own education. Meta-analysis with discrete-time event history models for 17 European countries (odds ratios and 95% confidence intervals are presented). |
| --- | --- |
|   *Note*: Controlled for gender, year childbearing union started, duration, and duration squared and parental separation. |   *Note*: Controlled for gender, year childbearing union started, duration, duration squared, parental separation and own education. |

| Figure A3. The association between the net effect of parental education and union dissolution, and the average crude divorce rate (without Russia as influential case). *b* = -0.053; *p* = .033. |
| --- |
|  |

*Note:* 1 = old union cohort (1970 – 1987), 2 = young union cohort (1988 – 2013)
